# Study of Ecological Relationship of Yeast Species with *Candida albicans* in the Context of Vulvovaginal Infections

**DOI:** 10.3390/microorganisms11102398

**Published:** 2023-09-26

**Authors:** Cátia Filipa Caetano, Carlos Gaspar, Ana Sofia Oliveira, Rita Palmeira-de-Oliveira, Lisa Rodrigues, Teresa Gonçalves, José Martinez-de-Oliveira, Ana Palmeira-de-Oliveira, Joana Rolo

**Affiliations:** 1 CICS-UBI—Health Sciences Research Center, Faculty of Health Sciences, University of Beira Interior, 6200-506 Covilhã, Portugal; catia.caetano@ubi.pt (C.F.C.);; 2 Faculty of Health Sciences, University of Beira Interior, 6200-506 Covilhã, Portugal; 3 Labfit-HPRD: Health Products Research and Development Lda, 6200-284 Covilhã, Portugal; 4 CNC-UC—Center for Neuroscience and Cell Biology, University of Coimbra, 3004-504 Coimbra, Portugal; 5 FMUC—Faculty of Medicine, University of Coimbra, 3004-504 Coimbra, Portugal

**Keywords:** biofilm, *Candida* spp., *Naganishia* spp., germ tube, *Malassezia* spp., *Rhodotorula* spp.

## Abstract

The role of the fungal community, the mycobiota, in the health of the vagina is currently an important area of research. The emergence of new sequencing technologies and advances in bioinformatics made possible the discovery of novel fungi inhabiting this niche. *Candida* spp. constitutes the most important group of opportunistic pathogenic fungi, being the most prevalent fungal species in vulvovaginal infections. However, fungi such as *Rhodotorula* spp., *Naganishia* spp. and *Malassezia* spp. have emerged as potential pathogens in this niche, and therefore it is clinically relevant to understand their ecological interaction with *Candida* spp. The main aim of this study was to evaluate the impact of yeasts on *Candida albicans*’ pathogenicity, focusing on in-vitro growth, and biofilm formation at different times of co-culture and germ tube formation. The assays were performed with isolated species or with co-cultures of *C. albicans* (ATCC10231) with one other yeast species: *Rhodotorula mucilaginosa* (DSM13621), *Malassezia furfur* (DSM6170) or *Naganishia albida* (DSM70215). The results showed that *M. furfur* creates a symbiotic relationship with *C. albicans*, enhancing the growth rate of the co-culture (149.69%), and of germ tube formation of *C. albicans* (119.8%) and inducing a higher amount of biofilm biomass of the co-culture, both when mixed (154.1%) and preformed (166.8%). As for the yeasts *R. mucilaginosa* and *N. albida*, the relationship is antagonistic (with a significant decrease in all assays), thus possibly repressing the mixture’s pathogenicity. These results shed light on the complex interactions between yeasts in the vaginal mycobiome.

## 1. Introduction

*Candida* species are one of the most prevalent opportunistic human fungal agents (85–95%) [1], and it is estimated that about 60% of healthy individuals are colonized by this yeast [2,3,4]. About 30% of women have vaginal colonization by this yeast, and in hospitals, it accounts for about 80% of documented fungal infections [1,5,6,7].

Of the more than 200 species belonging to this genus, one of the most reported to cause infections is *Candida albicans*, followed by other non-albicans *Candida* species, such as *Candida glabrata*, *Candida tropicalis*, *Candida parapsilosis*, and *Candida krusei* [8].

The ability of fungi, notably of the *Candida* genus, to colonize and infect various sites in the human body depends on their pathogenic factors [9,10]. Among these are morphogenesis, i.e., a transition from the yeast to a filamentous phase (the formation of germinative tubes), and biofilm formation [11]. In recent years, chronic infections caused by biofilm formation have been responsible for increased mortality rates, reaching more than 40% in 2020 [12].

In the vaginal mucosa, although much less abundant than bacteria, fungi communities, namely *C. albicans*, have been identified as one of the most dominant microorganisms that can affect vaginal health. Some studies have also shown that *Candida, Clavispora lusitaniae*, *Malassezia*, *Rhodotorula*, *Aspergillus*, and *Leptosphaerulina* are some of the most prevalent fungi in this niche [13].

In a recent study, we identified that the most prevalent species detected in clinical vaginal samples was *C. albicans*, present in 63.6% (21/33) of patients, followed by *Rhodotorula* spp. in 51.5% (17/33), *C. parapsilosis* in 15.2% (5/33), *Naganishia glabratus* in 9.1% (3/33), *C. tropicalis* in 3.0% (1/33), and *Saccharomyces cerevisiae* in 3.0% (1/33) [14]. Furthermore, *Rhodotorula* spp. and *C. parapsilosis* were more commonly associated with host colonization, while *C. albicans* were more associated with infection [14]. In another study, we found that *C. albicans* was the most prevalent *Candida* spp., both in acute and chronic cases of vaginal candidosis [15].

The interactions that occur between the microbial communities living in these niches have been implicated in the pathogenic process and may help to discover new antifungal therapies in the near future [16,17]. These interactions can occur in the form of a biofilm, with some studies already stating that *C. albicans* usually exists in the host as a heterogeneous biofilm with other microorganisms [18], and the infections caused by complex biofilm are about 30 to 60% and are called polymicrobial diseases [19]. Among the most studied microorganisms interacting with *C. albicans* are *Lactobacillus* spp. [20]. Bacteria such as *Lactobacillus fermentum* [21], *Streptococcus sanguinis* [22], and *Enterococcus faecalis* [23] can produce bacteriocins that inhibit the growth, dimorphic transition, biofilm formation, and virulence of *C. albicans*.

In the vast majority of studies already carried out, the interactions studied were essentially between *C. albicans* and some bacteria, which is why it is relevant to study these interactions between different fungal genera. One of the few fungi studied for its interaction with *C. albicans* and its potential use as a probiotic was *S. cerevisiae.* It was demonstrated that *S. cerevisiae* can inhibit *C. albicans* adherence to epithelial cells, its yeast-to-hypha switch, and the production of aspartyl proteases (SAP) [24]. So, the aim of this study was to assess possible ecological relationships between *R. mucilaginosa*, *N. albida* and *M. furfur* with *C. albicans*, through evaluation of pathogenicity of co-cultures with *C. albicans* in-vitro.

## 2. Methods

### 2.1. Strains

Collection strains from the American Type Culture Collection (ATCC) and the German Collection of Microorganisms and Cell Cultures (DSMZ) were used, namely *Candida albicans* ATCC 10231, *Rhodotorula mucilaginosa* DSM 13621, *Malassezia furfur* DSM 6170, and *Naganishia albida* DSM 70215. The cultures were kept at −80 °C in Brain Heart Infusion broth (BHI, VWR, Avantor, Radnor, PA, USA) supplemented with 20% glycerol (VWR, Avantor, Radnor, PA, USA). Yeast cultures were inoculated on Sabouraud Dextrose Agar (SDA, VWR, Avantor, Radnor, PA, USA) plates (for *C. albicans* and *R. mucilaginosa* and incubated at 37 °C and 25 °C, respectively), on Potato Dextrose Agar (PDA, Biolife, Monza, Italy) plates (for *N. albida* and incubated at 25 °C), and on SDA with 0.5% olive oil and 0.5% (*v*/*v*) Tween-80 (SDA supplemented) for *M. furfur* at 37 °C.

### 2.2. Determination of Growth Curves

Yeast cultures were inoculated into Yeast Peptone Dextrose broth (YPD, Fisher Scientific, Hudson, NH, USA). After incubation for 14h on an orbital shaker (Argitob 200 Aralab, Sintra, Portugal) an aliquot was taken and its optical density (O.D.) was read using an ultraviolet-visible spectrophotometer (UV-1700 PharmaSpec, Schimadzu Corp., Kyoto, Japan) and subsequently adjusted to an initial O.D. of 0.1. Hourly O.D. readings were repeated for a total of 48 h. With the readings of the optical densities, we carried out an analysis of the growth rate between 2 h, in the exponential phase, and its conversion into percentage, where 100% is the value of growth of the *C. albicans* strain. In addition to the readings performed in the spectrophotometer, serial dilution tests were carried out, where from each suspension, 20 μL are transferred to a 96-well plate, containing sterile Phosphate Buffer Saline (PBS: 1.37 M NaCl, Fisher Scientific, Hudson, NH, USA; 27 mM KCl, ChemLab, Zedelgem, Belgium; 100 mM Na_2_HPO_4_, Fisher Scientific, Hudson, NH, USA; 20 mM KH_2_PO_4_, ChemLab, Zedelgem, Belgium) (corresponding to the 10^−1^ dilution). Then, serial dilutions up to 10^−6^ were performed in PBS and the 10^−4^, 10^−5^, 10^−6^ dilutions were plated. For this purpose, 5 μL of each dilution was transferred in quadruplicate to SDA, PDA, and supplemented SDA plates (according to the microorganisms used). The plates were placed in the respective incubator overnight, after which the colony-forming units (CFU) were counted.

### 2.3. Biofilm Formation

The mixed biofilm assay aimed to evaluate the biofilm formed by yeasts in co-culture compared to that of *C. albicans*. Regarding the pre-formed biofilm, the purpose of this experiment was to understand if after the *C. albicans* was “installed” and in biofilm form, the addition of other yeasts would affect or not the already existing biofilm. In the laboratory, after incubation for 14 h at 25/37 °C in an orbital shaker and to study single biofilms formation, cells from the 4 yeasts in the study were collected by centrifugation (Hettich Zentrifugen, MiKRO 200R, Sigma-Aldrich, St. Louis, MO, USA) at 14,000× *g* for 2 min and washed twice with sterile PBS. The cells were then resuspended in a YPD culture medium at 0.5 MacFarland (1 × 10^6^ cells/mL), using a densitometer (Grant-bio, DEN-1, Grant Instruments, Fisher Scientific, Hudson, NH, USA). 100 μL of suspension was transferred to 96-well microplates and incubated for 48 h at 25/37 °C (Binder GmbH, Tuttlingen, Germany). To evaluate the capacity of the formation of mixed and preformed biofilms, the pre-inoculum, the washes with PBS and the suspensions were performed as explained above for the assay of simple biofilms. The mixed biofilms were performed as follows: 50 μL of *C. albicans* suspension was mixed with 50 μL of *N. albida* suspension, with 50 μL of *R. mucilaginosa* suspension or with 50 μL of *M. furfur* suspension. As control 50 μL of each yeast was added with 50 μL of YPD or YPD medium supplemented with 0.5% olive oil and 0.5% tween 80 (for *M. furfur*). The pre-formed biofilms were made from the pre-inoculum of *C. albicans* in YPD to a concentration of 0.5 MacFarland (1 × 10^6^ cells/mL) and transferred to a 96-well plate. 24 h later, a suspension was made from another pre-inoculum of *N. albida*, *R. mucilaginosa* and *M. furfur.* The biofilms formed with the *C. albicans* suspension were washed 3 times with PBS, and the inoculation of the 3 yeasts and their controls were added and revealed 24 h later.

### 2.4. Biofilm Biomass Quantification

The biofilm was fixed with 100 μL of 99% methanol (Fisher Scientific, Hudson, NH, USA) for 15 min and stained with 100 μL of 0.05% crystal violet (CV) (VWR, Avantor, Radnor, PA, USA) solution. The stained biofilm was resuspended with 33% (*v*/*v*) acetic acid solution. Before the plates were read at 590 nm, a 1:10 dilution with acetic acid (Fisher Scientific, Hudson, NH, USA) was performed. Culture medium was used as the negative control. This test was performed in two independent assays with four replicates each. At the same time, for both mixed and pre-formed biofilms, the CFUs were diluted and counted as was executed in the growth curves assay. The planktonic cells in these biofilms were removed by washing with sterile PBS. The pre-formed biofilms were also washed with PBS before the addition of the 2nd yeast suspension.

### 2.5. Biofilm Metabolic Viability

For metabolic activity, 100 μL MTT of 3-(4,5-dimethyl-thiazol-2-yl)-2,5-diphenyl tetrazolium bromide) solution (0.5 mg/mL) was added to each well [25]. The plates were covered and enveloped with aluminum foil and incubated in the dark at 25 °C and 37 °C for 4 h. Before reading the plates at 490 nm, a 1:10 dilution was made by placing 10 μL of solution from each well in a new well containing 90 μL of acetic acid.

### 2.6. Determination of the Ability to Form Germinative Tubes

To evaluate the percentage of germ tube formation of *C. albicans*, 125 μL of suspension (the suspensions were prepared in 2 mL of sterile PBS to a concentration of 1MacFarland) was transferred in 775 μL of YPD enriched with 10% Fetal Bovine Serum (Biochrom GmbH, Berlin, Germany) that was left in the shaker at 180 RPM for 2 h 30 min. The evaluation of the germ tube formation capacity of *C. albicans* in the presence of the other yeast species was performed by transferring 125 μL of the suspension of each yeast in 650 μL of YPD enriched with 10% Fetal Bovine Serum and incubated on an orbital shaker at 180 RPM for 2 h 30 min. After incubation, 10 μL of the culture was collected and observed under an optical microscope (YS100, Nikon, Tokyo, Japan) at 200× magnification, with a Neubauer’s chamber (Optik-Labor, Gorlitz, Germany).

### 2.7. Analysis of Results

The results for each isolate were expressed as the average of two independent assays with four replicates in each. To analyze the growth curves, the test used was the analysis of variance (one-way ANOVA), followed by the Kruskal–Wallis test. To analyze the difference in biofilm and germ tube formation between control, isolated yeasts and mixtures, the analysis of variance test (one-way ANOVA) was performed, followed by the Dunnett test with a confidence level of 95%. For the analysis of simple biofilms formed at 37 °C the *t*-student test was applied for statistical analysis. *p*-values equal to or less than 0.05 were considered statistically significant. The results were analyzed using GraphPad Prism 5 software.

## 3. Results

### 3.1. Growth Curves and Growth Rates

For the growth curve at 25 °C, we could observe that among the isolated yeasts the one that presents the highest growth exponential rate of growth during the 48 h is *R. mucilaginosa*, followed by *N. albida*. In relation to the co-cultures, *C. albicans* with *R. mucilaginosa* and *C. albicans* with *N. albida* presented growth curves with higher absorbance values, but lower growth rates (Table 1; 37% and 78%, respectively), when compared to *C. albicans* alone.

At the same time as the growth curves test, through the O.D. readings at 600 nm, we tried to understand the relation between the O.D. of the different yeasts and the value obtained in Colony Forming Units per milliliter (CFU/mL).

**Figure 1:**
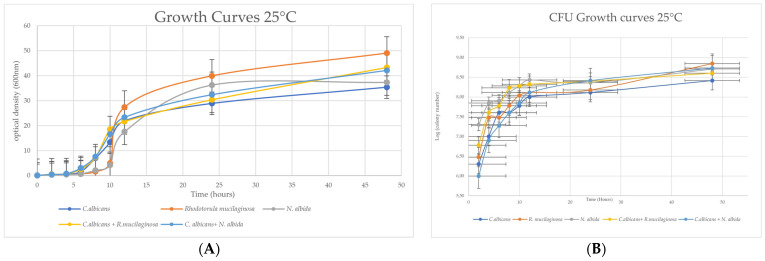
(**A**) Graphical representation of the optical density OD (600 nm) vs. Time (hours) of the 48-h growth curve of the strains *C. albicans*, *R. mucilaginosa*, and *N. albida* and the mixtures of the two last yeasts with *C. albicans*; (**B**) Graphical representation of the log (CFU/mL) vs. Time (in hours) of the growth curve of the three yeasts isolated and the mixtures of *C. albicans* + *R. mucilaginosa* and *C. albicans* + *N. albida*.

Through Figure 1B, we can verify that there is a direct relationship between the different curves. For the mixture constituted by the yeasts at 25 °C the results of both curves coincide, regarding the increase in its growth when compared to the one of *C. albicans.*

In addition to the two tested temperatures, we also looked for the possible influence of the liquid medium that may preferentially benefit the growth of the *C. albicans* strain (YPD) or of *M. furfur* (YPD supplemented with fatty acids).

**Figure 2:**
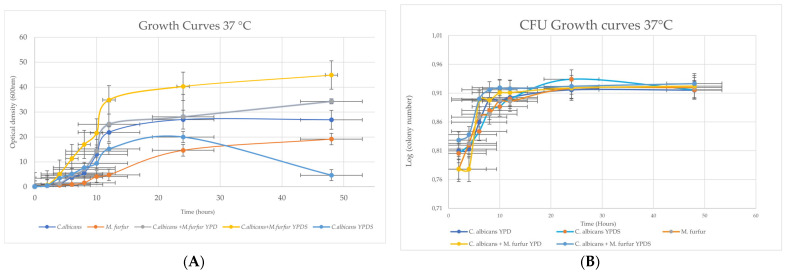
(**A**) Graphical representation of the optical density (O.D. 600 nm) vs. Time (in hours) of the growth curve of *C. albicans* and *M. furfur* strains and of the mixtures under study in two different liquid media (YPD and supplemented YPD). (**B**) Graphical representation of the log (CFU/mL) vs. Time (in hours) of the growth curve of the two yeasts isolated and the mixtures in YPD and supplemented YPD (YPDS).

Therefore, in Figure 2A, we show the yeast growth curves at 37 °C and the mixtures with the two different culture media. It was observed that there is a noticeable difference between *C. albicans* when compared to the mixtures (either in YPD or YPD medium with lipid supplementation). This increase was more pronounced for the mixture of YPD with lipid supplementation, as we can see from the growth rate calculated between two points on the curve (Table 2). In Table 3, we can observe that, when compared to *C. albicans* in supplemented YPD, the growth of the mixtures is even more visible. In addition to the differences present in the growth rate, we also observed that when these yeasts are together, they enter the exponential phase sooner, than when we have *C. albicans* and *M. furfur* alone. We also can see that *C. albicans* grows slower in the YPDS medium, and in fact, the yeast begins to enter the death phase around 24 h. Therefore, we can see that the culture medium YPDS has no influence on the results obtained for the mixture of *C. albicans* and *M. furfur*. The increase in growth of this mixture possibly comes from the interaction with *M. furfur*.

Contrasting with the results in Figure 2A showing that the mixtures had a much higher growth rate than *C. albicans*, in Figure 2B this is not the case. There is no noticeable difference between *C. albicans* and *M. furfur* yeasts and their mixture.

### 3.2. Biofilms

**Figure 3:**
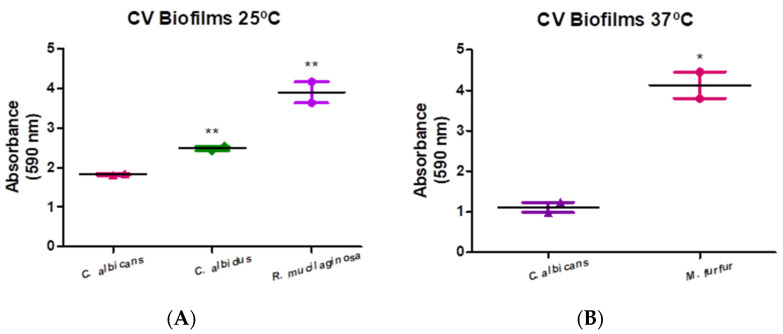
Graphic representation of biofilm formation: (**A**) at 25 °C by *C. albicans*, *R. mucilaginosa*, and *N. albida* at 48 h of incubation and respective standard errors. (**B**) at 37 °C by *C. albicans* and *M. furfur* at 48 h of incubation and their respective standard errors. For the analysis of simple biofilms formed at 37 °C the *t*-student test was applied, the analysis of simple biofilms at 25 °C the One-way ANOVA test was performed, followed by the Dunnett test with a confidence level of 95%. Values of *p* < 0.05 were considered statistically significant (represented by the symbol *—one significance level and **—two levels of significance).

All strains used in this study can produce biofilm (Figure 3). Compared with the biofilm formed by *C. albicans* at 25 °C, the strain that produced the highest biofilm biomass was *R. mucilaginosa* (213.41%), followed by *N*. *albida* (135.98%). At 37 °C, *M. furfur* was the yeast that produced more biofilm (371.01%).

### 3.3. Mixed Biofilms

**Figure 4:**
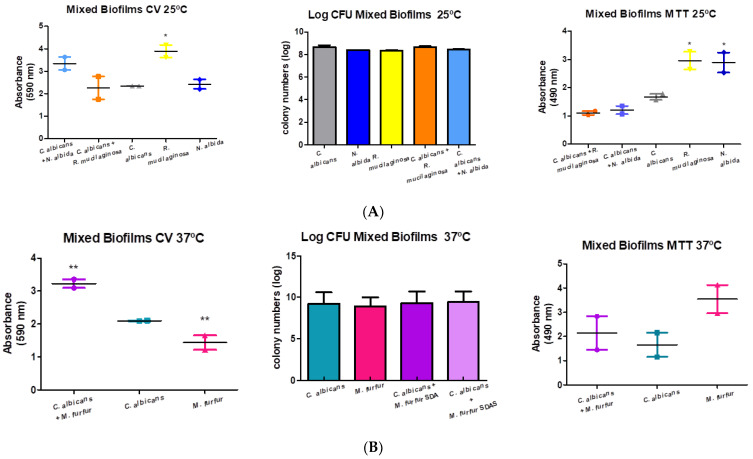
Graphical representation of the formation of mixed biofilms: (**A**) at 25 °C of the three ATCC isolates, and the mixtures carried out by *C. albicans* + *R. mucilaginosa* and *C. albicans* + *N. albida*; (**B**) at 37 °C of the two ATCC isolates, and the mixture carried out by *C. albicans* + *M. furfur*. One-way ANOVA test was performed, followed by the Dunnett test with a confidence level of 95%. Values of *p* < 0.05 were considered statistically significant (represented by the symbol *—one significance level and **—two levels of significance).

The results for mixed biofilms are presented in Figure 4. Figure 4A shows an increase in the production of total biomass with the mixture of *C. albicans* and *N. albida* (142.89%). However, this result was controversial to that presented by the CFU/mL, which did not reveal variations in the number of colonies.

The mixture of *C. albicans* and *R. mucilaginosa* did not show relevant differences in the biofilm formed when compared to the control (96.46%). The results of metabolic activity showed a decrease (Dunnett test: not statistically significant) for both mixtures (72.28% and 65.87%, respectively).

Figure 4B shows statistically significant results for the mixture of *C. albicans* and *M. furfur*. This mixture showed higher values of both total biomass production (154.06%, Dunnett test: *p*-value = 0.0075) and metabolic activity (129.39%, Dunnett test: *p*-value = 0.2118). Therefore, the increase in biomass was greater than the increase in metabolic activity, in this particular interaction. The CFU/mL did not reveal very relevant differences between the isolates and the mixture.

### 3.4. Preformed Biofilms

**Figure 5:**
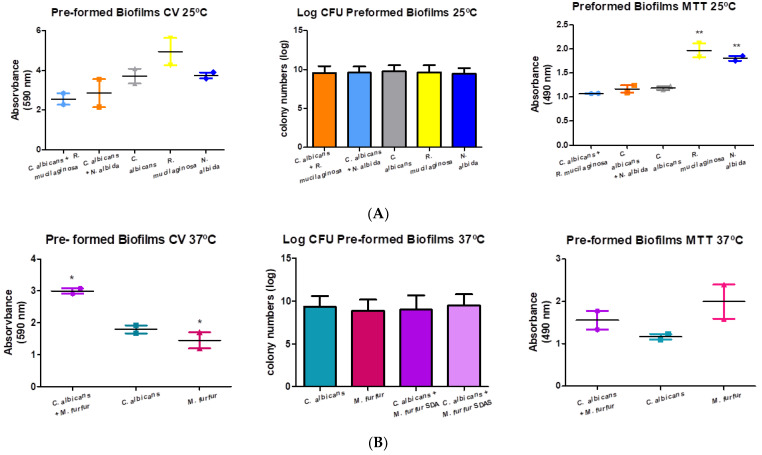
Pre-formed biofilm formation by crystal violet (590 nm), log (CFU/mL), and MTT (490 nm) assay. One-way ANOVA test was performed, followed by the Dunnett test with a confidence level of 95%. Values of *p* < 0.05 were considered statistically significant (**A**) at 25 °C and (**B**) at 37 °C (represented by the symbol *—one significance level and **—two levels of significance).

In Figure 5A we can observe that there was a decrease (Dunnett test: not significant), in the absorbance values registered for the mixtures in relation to the control (76.90% for *N. albida* and 69.02% for *R. mucilaginosa*). Regarding the metabolic activity, the obtained results for the co-cultures did not show any differences compared to the result of *C. albicans* (90.32% and 98.53%, respectively).

The evaluation of the total biomass was shown to be higher (166.85%, Dunnett test: *p*-value = 0.0151), and statistically significant, for the *C. albicans* and *M. furfur* co-culture. Regarding the metabolic activity, the co-culture obtained higher absorbance results (133.48%, Dunnett test: *p*-value = 0.2397) when compared to *C. albicans* (Figure 5B).

### 3.5. Germ Tube

For *C. albicans* one of the most important virulence factors is its ability to form germ tubes, that is, its ability to transition from the yeast form to the hyphal form. To carry out a comparison between the mixtures of isolates and *C. albicans*, and although it is already known that 37 °C corresponds to the optimal temperature for the formation of a high number of germ tubes, we tested the results also at 25 °C.

**Figure 6:**
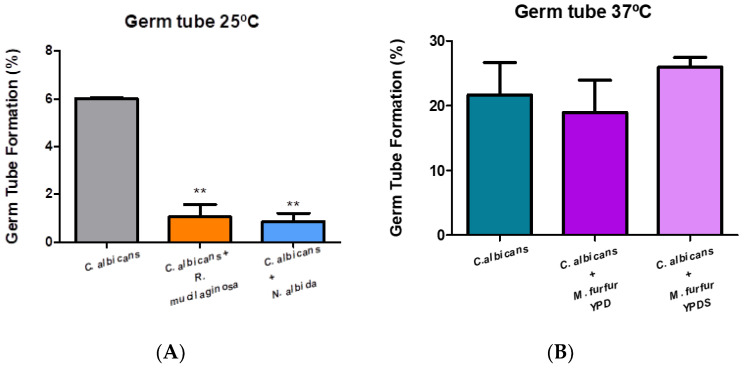
Evaluation of the percentage of germ tube formation of *C. albicans* after 2 h 30 min in the orbital shaker: (**A**) at 25 °C and (**B**) at 37 °C and the corresponding co-cultures. One-way ANOVA test was performed, followed by the Dunnett test with a confidence level of 95%. Values of *p* < 0.05 were considered statistically significant by the One-way Anova Test (represented by the symbol **).

Figure 6A shows that, when mixed, the capacity of yeasts to form germ tubes decreases considerably. The difference in the capacity to form germ tubes was statistically significant for both mixtures of isolates when compared with the control (Dunnett test: *p*-value = 0.0034)). According to Figure 6B, we can visualize that the isolates at 37 °C when mixed in YPD medium slightly decrease (Dunnett test: *p*-value = 0.5552) the number of germ tubes (%) formed. In contrast, the mixture with the supplemented YPD medium showed a slight increase in relation to the tubes produced by *C. albicans*. The difference in germ tube forming capacity was not statistically significant for both mixtures of the isolates when compared with the control (*p* > 0.05).

### 3.6. Overview Table

To facilitate the analysis and draw conclusions, we present a summary table where the results mentioned above are all included. This table represents the tests performed and the results of the mixtures only and not of their isolated actions. The arrows represent the effect of the mixture in the control, having three different colors. The green color represents an increase in the virulence of *C. albicans* in the presence of another yeast, the orange color a slight decrease in the results when compared with the isolated action and the red color shows a decrease in the action of *C. albicans* in co-culture with another yeast.

In this illustration of results, we can say that the yeasts *R. mucilaginosa* and *N. albida* when co-existent with *C. albicans* lead to a decrease in pathogenicity and virulence of the overall mixture. For *M. furfur*, the conclusion is totally opposite, since the junction of both yeasts will contribute to an increase in the number of cells, adherence, and invasion of the host by the microorganisms. The two arrows in Table 4 represent the results of the mixed biofilms of *C. albicans* and *N. albida*, where there was an increase in total biomass but a decrease in metabolic activity. These results may have been due to the fact that the biomass is a result of a mixture of both viable and non-viable cells, unlike the MTT test, in which only viable cells are counted.

Therefore, we can say that the two yeasts that exert an inhibitory effect on the action of *C. albicans* can be classified as antagonists. *M. furfur,* on the other hand, can be designated as a symbiont with *C. albicans*, because when together they grow and multiply and increase their virulence factors.

## 4. Discussion

Recent studies have shown that fungal diseases have a high mortality rate, estimated to kill more than 1.6 million people every year [26]. Alterations in the balance of the vaginal mycobiome can cause pathogens to grow exponentially, leading to more complex vaginal infections [27]. Vulvovaginal candidiasis (VVC) is one of the most common infections and it is estimated that about 75% of women have been infected at least once [28,29,30]. It is known that the colonization, survival, and growth of pathogenic fungi in the host, as well as the damage caused to the host, are highly dependent on virulence factors [31].

In this work we evaluated the ecological relationship of *C. albicans* with three emerging fungal genera of the vaginal niche, focusing on growth curves, biofilm and germ tube forming capacity of the different co-cultures.

We first evaluated the growth of the strains in isolation. The results between the growth curves with the absorbance readings and the curves with the colony counts differ, due to the fact that the colony count is a much more sensitive method. Researcher errors, the appearance of countless colonies and other factors contribute to this sensitivity. In addition, only viable microorganisms are counted, unlike absorbance readings. We observed that the growth of *R. mucilaginosa* is higher than that of *N. albida*, followed by *C. albicans.* At 37 °C we observe that the growth of *M. furfur* showed a lower exponential rate growth than *C. albicans*. Of the mixtures at 25 °C, the one that stands out for the most abrupt growth decrease is the mixture constituted by *C. albicans* with *R. mucilaginosa.* These results may indicate the presence of some metabolite in the culture medium, which probably acts on the fungal cells to inhibit their growth. During the growth of *C. albicans*, it synthesizes some metabolites, such as fatty acids, and in the exponential phase, due to the increase in iron, Fum 12 (fumarate hydratase) is synthesized [32]. A study reported that *Rhodotorula* spp. can produce glycogen during the exponential phase and is able to accumulate lipids and carotenoids during the stationary phase [33]. A further analysis of the metabolites of the emerging yeasts and their function present in the supernatants of the yeasts still needs to be concretized.

The growth curve at 37 °C showed that in the mixture with *M. furfur* on YPD medium, the growth is higher (122%) compared to *C. albicans*. However, in the YPD medium supplemented the growth is even higher (150%). This increase in growth may be due to the fact that *Malassezia* spp., being a lipo-dependent yeast, may exhibit a failure in the production of myristic acid, which is a precursor of fatty acids [34]. The necessity of fatty acids, so that its growth occurs, makes us believe that, as *C. albicans* can produce fatty acids between the latent and exponential phases, it will stimulate *M. furfur* to grow due to the increased nutrients available. The metabolism of *Malassezia* spp. possesses the ability to produce catalase, urease, and β-glucoside and during the exponential phase produces lipases. Therefore, these yeasts, when present at the same time, will increase their growth rate and their number of viable cells capable of colonizing and will therefore be able to cause infection in the host.

In recent decades, the incidence of fungal infections due to biofilm formation has reached 65% of all recorded cases [35]. The results of the mono-species biofilms showed that, in relation to *C. albicans*, all the other isolates showed higher biomass values, with statistically significant differences (Dunnett test: *p*-value = 0.0059). These results agree with the literature, which states that all isolates are specially adapted to grow on surfaces being excellent candidates as biofilm formers [36,37,38]. However, this aspect is not so well studied for all yeast genera, when compared with the studies already executed in bacteria.

In the case of biofilm formation by yeasts, researchers state that the different results found, suffer alterations due to factors such as the culture medium used, the availability of oxygen and nutrients, the pH and the origin of the species [39]. For *Candida* spp., the formation of hyphae (important for biofilm maturation to occur followed by dispersion) can contribute to finding higher amounts of total biomass [40]. Some studies state that when compared between species of this genus, *R. mucilaginosa* was the one that showed a greater ability to form biofilm [41].

The capacity to form a biofilm with the yeasts in co-culture was also evaluated. When counting biofilm colonies, we were unable to distinguish between the colonies of the different yeasts, which was one of the limiting factors of our study. For further work, it would be important to continue this study and deepen knowledge already obtained, through the use of fluorescent probes or selective solid media so that it is possible to distinguish colonies of different genera when counting colony-forming units. The results showed that in the mixtures with *R. mucilaginosa* the amount of biofilm formed was lower (Dunnett test: no statistical significance when compared with the control). Studies carried out in 2019, state that it may exist in *Rhodotorula* spp. a probiotic effect. These findings were found after comparison with *Saccharomyces* spp. These effects occur due to competition with the intestinal flora and the binding of pathogens or toxins to the cell surface of these yeasts [42,43]. What is thought is that *Rhodotorula* spp. expresses similar molecules in its cell wall, and therefore, it is expected to exert beneficial effects by decreasing inflammation [42,43]. Nevertheless, the effect may be less evident than in *Saccharomyces* spp., since *Rhodotorula* spp. do not have 1-3-β-glucan in their cell wall and therefore will not be able to activate the dectin 1 receptor on the surface of macrophages, consequently failing to stimulate the local immune system [44,45]. This leads us to consider that our results may come from some probiotic effect of *Rhodotorula* spp. More studies are needed for a better understanding of this entire mechanism.

As for *N. albida*, the biomass values of its mixture with *C. albicans* were higher (Dunnett test: no statistical significance) than the isolated control, but the metabolic activity values revealed a decrease (Dunnett test: no statistical significance) in cellular metabolism. Although the total biomass values indicated an increase in the amount of biofilm produced by this mixture, the CFU/mL showed a decrease (Dunnett test: no statistical significance) in colon formation compared to *C. albicans*. This may lead us to think that the reading of the total biomass, as it is a reading of viable, dead cells and extracellular matrix, led to a misreading of the fungal mass. For the mixture with *M. furfur*, there was a significant increase in the total biomass and an increase in cell viability. With these results, we can conclude that *C. albicans* and *M. furfur* when mixed, form more biofilm, become more viable and, therefore, can become more virulent causing more infection and also a more difficult infection to cure. Some previous studies have demonstrated that the biofilm-forming capacity of *Candida* spp. and *Malassezia* spp. are dependent on a few factors, such as hydrophobicity, adherence, and phospholipase production, which may help explain the dimorphic transition from commensal to pathogenic of these microorganisms [46]. Hydrophobicity has a major contribution to microbial adhesion, increasing hydrophobic interactions, allowing the microorganism to form biofilm, and increasing their drug resistance and virulence [47]. The results of the pre-formed biofilms showed interesting results. For the mixtures at 25 °C, both showed a decrease (Dunnett test: no statistical significance) in the ability to form biofilm. We can also see that the results obtained for *C. albicans* in the mixed and pre-formed biofilms show different absorbance values. The increase in the pre-formed biofilms may be due to the fact that the suspension was added 24 h before the reading, unlike the mixed biofilm, which was revealed 48 h later. For the mixture of *C. albicans* with *M. furfur* there was a significant increase in the amount of biofilm formed. In the evaluation of the metabolic activity for the isolates at 25 °C, both mixtures showed no difference in cell viability. In the mixture at 37 °C there was a non-significant increase in cell viability when compared with *C. albicans* isolate.

In addition to the ability to form biofilm, other factors are very important to trigger yeast infections. One of these is the formation of the germ tube. The ability to differentiate the two forms is directly related to the invasion of host tissues, facilitating their penetration [48]. In all studies, the optimal temperature for germ tube formation of *C. albicans* is 37 °C, however, as we are working with yeasts that cannot grow at such high temperatures, we carry out the control (isolated *C. albicans*) for the two temperatures under study. As we expected, there was a difference from the two controls where at a temperature of 37 °C our strain formed approximately 22% of germ tubes. In relation to the mixtures at 25 °C, we can observe that there was a significant decrease (Dunnett test: *p*-value = 0.0034) in germ tube formation. In the case of *R. mucilaginosa*, a study in 2019 highlighted the absence or rare ability to form pseudohyphae or hyphae [49].

Of the three yeasts under study, *R. mucilaginosa* (due to its possible probiotic action), and *M. furfur* may, with future trials, help researchers discover new treatments and diagnostic methods for vulvovaginal infections. *N. albida* seems to have no influence on the virulence of *C. albicans*. This study had some limitations, with no results showing which of the yeasts exert more influence on the mixtures, having only raised hypotheses according to the morphological, biochemical and virulence characteristics found in scientific articles.

## 5. Conclusions

Currently, studies on the vaginal mycobiota have been investigated with more emphasis, namely the presence of important yeasts in this niche. However, the role of different fungal genera in vaginal health remains to be investigated, and so, in this work, we intend to explore the ecological dynamics of these species in simulated co-habitation in a vaginal environment.

Therefore, this study aimed to evaluate the ecological relationship of the three selected emerging yeasts when present in co-culture with *C. albicans*. It was found that for the mixtures, *C. albicans* with *R. mucilaginosa* and *C. albicans* with *N. albida*, showed a decrease in the expression of the virulence phenotype in all assays. This decrease in the virulence of the yeast co-culture was more pronounced for the mixture of *C. albicans* with *R. mucilaginosa*. For the mixture of *C. albicans* with *M. furfur,* it was found that there was an increase in all assays, being translated into a possible symbiotic relationship between the two yeasts, increasing the pathogenicity when in co-culture, that will lead to an increase in the occurrence of infection.

In addition, in the future, more tests are needed in the field of vaginal mycology. Researchers will be able to know better functions and/or the consequences that these genera can bring to vaginal health, managing to develop antifungal therapies with a greater capacity to prevent colonization and installation of the disease.

## Figures and Tables

**Table 1:** Calculation of the growth rate of yeasts at 25 °C and the two corresponding mixtures during the exponential phase of the microbial curve (at t10 and t12) and calculation of the corresponding percentage growth compared to the *C. albicans* control (100%). One-way ANOVA test was performed, followed by the Kruskal–Wallis test. Values of *p* < 0.05 were considered statistically significant. The results were not statistically significant (*p*-value = 0.4060).

Yeasts	Growth Rates	%
*C. albicans*	0.071 ± 2.660	100.00
*R. mucilaginosa*	0.187 ± 1.185	262.76
*N. albida*	0.112 ± 0.903	157.20
*C. albicans + R. mucilaginosa*	0.027 ± 1.460	37.24
*C. albicans + N. albida*	0.056 ± 2.538	78.39

**Table 2:** Calculation of the growth rate of yeasts at 37 °C and the corresponding mixtures during the exponential phase (at t10 and t12) and calculation of the corresponding percentage growth compared to the *C. albicans* YPD control (100%). One-way ANOVA test was performed, followed by the Kruskal–Wallis test. Values of *p* < 0.05 were considered statistically significant. The results were not statistically significant (*p*-value = 0.3916).

Yeasts	Growth Rates	%
*C. albicans* YPD	0.074 ± 3.703	100.00
*M. furfur*	0.006 ± 1.228	7.73
*C. albicans + M. furfur* YPD	0.090 ± 6.895	121.73
*C. albicans + M. furfur* YPDS	0.111 ± 14.32	149.69

**Table 3:** Calculation of the growth rate of yeasts at 37 °C and the corresponding mixtures during the exponential phase (at t10 and t12) and calculation of the corresponding percentage growth compared to the *C. albicans* YPDS control (100%). One-way ANOVA test was performed, followed by the Kruskal–Wallis test. Values of *p* < 0.05 were considered statistically significant. The results were not statistically significant (*p*-value = 0.3916).

Yeasts	Growth Rates	%
*C. albicans* YPDS	0.048 ± 0.6955	100.00
*M. furfur*	0.006 ± 1.228	11.87
*C. albicans + M. furfur* YPD	0.090 ± 6.895	186.92
*C. albicans + M. furfur* YPDS	0.111 ± 14.32	229.88

**Table 4:** Summary table of all the tests performed in this work and their results. The arrows represent the effect of the mixture in the control. The green color represents an increase in the virulence of the yeast mixture, the orange color a slight decrease in the results and the red color shows a decrease in the virulence of the yeast mixture.

*C. albicans* +	*R. mucilaginosa*	*M. furfur*	*N. albida*
Growth Curves	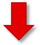	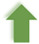	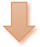
Mixed Biofilms	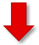	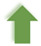	Biomass 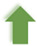 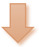 Metabolic activity
Preformed Biofilms	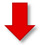	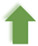	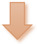
Germ Tube	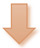	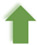	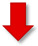

## Data Availability

The data presented in this study is available within the article.
